# VGLUT2 and APP family: unraveling the neurobiochemical mechanisms of neurostimulation therapy to STZ-induced diabetes and neuropathy

**DOI:** 10.3389/fendo.2024.1336854

**Published:** 2024-02-02

**Authors:** Yitong Zhang, Chenxuan Wu, Wenqi Jiang, Yan Cao, Dongtai Chen

**Affiliations:** ^1^ School of Medical Technology, Beijing Institute of Technology, Beijing, China; ^2^ School of Life Science, Beijing Institute of Technology, Beijing, China; ^3^ State Key Laboratory of Oncology in South China, Guangdong Provincial Clinical Research Center for Cancer, Sun Yat-sen University Cancer Center, Guangzhou, China

**Keywords:** DPN, neurostimulation, VGLUT2, amyloid precursor protein family, APP, APLP1, APLP2

## Abstract

Diabetic Peripheral Neuropathy (DPN) poses an escalating threat to public health, profoundly impacting well-being and quality of life. Despite its rising prevalence, the pathogenesis of DPN remains enigmatic, and existing clinical interventions fall short of achieving meaningful reversals of the condition. Notably, neurostimulation techniques have shown promising efficacy in alleviating DPN symptoms, underscoring the imperative to elucidate the neurobiochemical mechanisms underlying DPN. This study employs an integrated multi-omics approach to explore DPN and its response to neurostimulation therapy. Our investigation unveiled a distinctive pattern of vesicular glutamate transporter 2 (VGLUT2) expression in DPN, rigorously confirmed through qPCR and Western blot analyses in DPN C57 mouse model induced by intraperitoneal Streptozotocin (STZ) injection. Additionally, combining microarray and qPCR methodologies, we revealed and substantiated variations in the expression of the Amyloid Precursor Protein (APP) family in STZ-induced DPN mice. Analyzing the transcriptomic dataset generated from neurostimulation therapy for DPN, we intricately explored the differential expression patterns of VGLUT2 and APPs. Through correlation analysis, protein-protein interaction predictions, and functional enrichment analyses, we predicted the key biological processes involving VGLUT2 and the APP family in the pathogenesis of DPN and during neurostimulation therapy. This comprehensive study not only advances our understanding of the pathogenesis of DPN but also provides a theoretical foundation for innovative strategies in neurostimulation therapy for DPN. The integration of multi-omics data facilitates a holistic view of the molecular intricacies of DPN, paving the way for more targeted and effective therapeutic interventions.

## 1 Introduction

Diabetic peripheral neuropathy (DPN), the most prevalent vascular complication of diabetes, exhibits an escalating incidence that profoundly jeopardizes health and quality of life (1). Despite its prevalence, the pathogenesis of DPN remains elusive. Existing research suggests that the formation of glycosylation end products, hyperglycemia, inflammatory responses, oxidative stress, and nerve growth factor collectively contribute to the mechanisms underlying DPN (2). Current therapeutic strategies for DPN primarily concentrate on stringent blood glucose control, dietary management, and the administration of drugs aimed at enhancing nutrition, promoting repair, combating oxidative stress, and improving microcirculation (3). However, clinical outcomes indicate a lack of treatments capable of effectively reversing DPN.

In recent years, neuro-electrophysiological investigations have drawn attention to the impact of acupuncture on DPN. Electroacupuncture, in particular, offers advantages in terms of stability, continuity, and adjustability compared to traditional acupuncture therapy (4). It has demonstrated notable clinical efficacy in alleviating symptoms and enhancing neurological function, findings substantiated by multiple studies (5). Consequently, there exists a pressing need to delve into the molecular pathogenesis of DPN and explore the potential of this novel therapeutic approach.

Vesicular glutamate transporters (VGLUT) are situated on the vesicular plasma membrane of presynaptic neurons within glutamatergic cells, facilitating the specific transport of glutamate from the cytoplasm into synaptic vesicles (6). The rate and extent of glutamate transport into vesicles are contingent upon factors such as the number of vesicles and extracellular glutamate concentration (7). This process plays a crucial role in the pathogenesis of various diseases, including diabetes. Notably, VGLUT2 predominately manifests in the diencephalon and brainstem of the central nervous system. A prior investigation has validated the participation of VGLUT2 in the transportation of glutamate during the insulin secretion process in pancreatic cells, implicating VGLUT2 in β cell apoptosis and, consequently, the onset of diabetes (8).

Remarkably, VGLUT2-expressing neurons contribute to transmitting peripheral pain signals to the spinal cord. Activation of glutamatergic dorsal horn neurons expressing VGLUT2 leads to a reduction in mechanical and thermal withdrawal thresholds (9). However, the precise role of VGLUT2 in the genesis and progression of DPN remains elusive and warrants further exploration.

Amyloid precursor protein (APP) is a transmembrane protein characterized by a substantial N-terminal extracellular domain and a compact C-terminal cytoplasmic domain. Widely distributed across the central nervous system, liver, adipose tissue, and other organs, APP assumes a crucial role in cellular adhesion and neurodevelopment (10). Its established association with Alzheimer’s disease has been extensively documented in previous research (11). Beyond this, APP is implicated in kinesin-mediated vesicular transport, potentially encompassing neurotransmitter vesicles.

Short-term processing of APP has been identified to correlate with an augmentation in glutamate release, a trend similarly observed in long-term processing. Throughout the progression of Alzheimer’s disease, the synaptic transmission-expressed VGLUT2 protein demonstrates downregulation in the absence of both APP and amyloid precursor-like protein 2 (APLP2) (12). Additionally, APP and APLP2 play indispensable roles in regulating the expression of VGLUT2 during neural differentiation (13). These findings offer a theoretical foundation for exploring a novel APP/VGLUT2 mechanism in the context of DPN therapy.

In this study, our investigation involved comprehensive data mining across multiple omics datasets, revealing differential expression of VGLUT2 at both transcriptional and translational levels in DPN. To substantiate these findings, we employed a DPN mouse model constructed through intraperitoneal injection of Streptozotocin (STZ) and validated the results of the differential expression analysis using qPCR and Western blot. Furthermore, our gene expression analysis, conducted through a combination of microarray and qPCR, identified and verified differential expression of the APP protein family in the context of DPN. We found that the protein and mRNA level of VGLUT2 were up-regulated in spinal dorsal horn of STZ-induced DPN mice, and mRNA levels of APP, APLP1 and APLP2 were also up-regulated. Within the high-throughput dataset generated from neurostimulation therapy for DPN, we conducted analysis of the differential expression of VGLUT2 and APPs, along with correlation analysis, aiming to elucidate molecular mechanisms underlying neurostimulation therapy for DPN. Finally, utilizing protein-protein interaction predictions and functional enrichment analyses, we forecasted the involvement of VGLUT2 and APPs in the biological processes associated with the onset of DPN and during the course of neurostimulation therapy.

## 2 Materials and methods

### 2.1 Animals

C57BL/6J male mice aged 5-6 weeks were obtained from the Guangdong Medical Laboratory Animal Centre in Guangzhou, China, with an average weight of 18–22 g (n = 3-6). The experimental protocol was thoroughly reviewed and approved by the Sun Yat-sen University Cancer Centre Animal Care and Use Committee (Sun Yat-sen University No.: L025501202205002). Euthanasia of the animals was carried out using the carbon dioxide (CO_2_) asphyxiation procedure.

### 2.2 Study design

To induce DPN in mice, male C57BL/6J mice (5-6 weeks old) were administered STZ (50 mg/kg, i.p.) once a day for four consecutive injections. For the preparation of STZ, 2.1g of citric acid (FW: 210.14) was dissolved in 100mL of double-distilled water to form liquid A and 2.94g of sodium citrate (FW: 294.10) was dissolved in 100mL of double-distilled water to form liquid B. When required, liquids A and B were mixed in a specific ratio (1:1.32 to 1:1), and the pH value was adjusted to 4.2-4.5 using a pH meter, producing the citric acid buffer for STZ preparation.

Control mice received only i.p. injections of the vehicle solution (citric acid buffer, pH 4.5). All STZ-injected mice exhibited elevated blood glucose levels (> 16.7 mmol/L). The successful establishment of DPN was confirmed at 21 days post-injections through Von-Frey testing and the Hot Plate Test.

### 2.3 Fasting blood glucose (FBG) and random blood glucose (RBG) test

To measure blood glucose levels, blood was taken from 1 mm of clipped tail of mice. FBG and RBG were measured with a glucometer and disposable test strips (One Touch Lifescan, Malvern, PA, USA). The mice were fasted overnight (16 h) before the start of testing FBG. Mice are considered to have hyperglycemia, FBG with a single blood glucose measurement of > 150 mg/dl, and RBG with two consecutive levels measurement value > 250 mg/dl.

### 2.4 Mechanical hyperalgesia—Von-Frey test

The paw withdrawal threshold (PWT) of mice was assessed using the Von-Frey filament, employing the ‘up and down’ method, with detailed procedures outlined in a prior study (14). Each mouse underwent an adaptive test three days prior to the formal experiment, lasting for one hour. Positive reactions, such as hind paw retraction, movement, or lameness, were recorded along with their corresponding values. The mechanical threshold is expressed as the logarithm (base 10) of the diameter sensitivity (ds) [log10(10 * force in milligrams)].

### 2.5 Hot plate test

The paw withdrawal latency (PWL) of mice was assessed using a hot plate apparatus set at 55 °C (ZH-6C, ANHUI ZHENHUA BIOLOGIC APPARATUS FACILITIES, China), following established procedures detailed in a prior study (15). Prior to the experiment, mice underwent a three-day habituation period. The test was conducted in triplicate, with a minimum interval of 5 minutes between each trial, and the average PWL values were calculated for each mouse.

### 2.6 Real-time qPCR

RNA extraction from spinal cord tissues of mice with Paclitaxel-Induced Peripheral Neuropathy was performed using a Tissue RNA Purification Kit Plus (ESscience, China) following the provided instructions. Subsequently, cDNA synthesis was carried out using the EZBioscience Color Reverse Transcription Kit (Roseville, USA). For quantitative real-time polymerase chain reaction (qRT-PCR), the SYBR Green qPCR Super Mix (EZBioscience, Roseville, USA) and the A CFX96 Touch Real-Time PCR System (Bio-Rad, USA) were employed to run the thermal cycling program, and data were analyzed using the comparative threshold cycle (Ct) method. The primer pairs utilized for qRT-PCR are as follows:

APP: Forward - TCCGTGTGATCTACGAGCGCAT, Reverse – GCCAAGACATCGTCGGAGTA GT. APLP1: Forward - AGGAGCGTATGGACCAGTGTGA, Reverse – TACTCCACACCTCGGA ACCGAT. APLP2: Forward - AGAAGCCATGCTGAATGACCGC, Reverse – GGCGATCTTTGT TCTCAGCACG. VGLUT2: Forward - CCTATGCTGGAGCAGTCATTGC, Reverse – GGCTCTC ATAAGACACCAGAAGC. GAPDH: Forward - CATCACTGCCACCCAGAAGACTG, Reverse – ATGCCAGTGAGCTTCCCGTTCAAG.

### 2.7 Western blot analysis

In accordance with previously established protocols (16), total proteins from the spinal cord were lysed using RIPA buffer, supplemented with protease and phosphatase inhibitors (RIPA: PMSF: phosphatase inhibitor = 100:1:1). The lysates were separated by SDS–PAGE and subsequently transferred to polyvinylidene fluoride (PVDF) membranes (0.22 μm, Pall, USA). Blocking was conducted in TBST containing 5% non-fat dry milk. Detailed information regarding the antibodies used in both the primary and secondary stages of the Western blot assay is provided. Protein visualization was achieved through detection with AMER sham ECL Western blotting detection reagents (GE Healthcare).

### 2.8 Data collection

The mRNA expression data pertaining to DPN were sourced from the Gene Expression Omnibus (GEO) database (http://www.ncbi.nlm.nih.gov/geo/), specifically from datasets GSE95849, GSE34889, GSE147732, and GSE70852. To access gene expression data relevant to treated DPN, we consulted the GEO Series GSE155741 and GSE156184.

GSE95849 using microarray-based genome-wide expression analyses, identified the pathogenesis of DM and DPN based on the samples from the health records and blood samples (2 mL) of the participants. There were 3 groups of control, diabetes and DPN, including 6 samples in each group.

GSE34889 showed the transcription profiling for the sciatic nerve of 4 groups of mice: (8 week old db/db (n = 8) or db/+ (n = 8), and 24 week old db/db (n = 6) or db/+ (n = 7)). 8 weeks and 24 weeks represented the early and advanced stages of DPN. C57BLKS/J background, Cg-m+/+Lepr db/J (BKS-db/db) mice.

Diabetes in GSE147732 was induced with a single intraperitoneal injection of STZ (55mg/kg); control rats were performed with a single intraperitoneal injection of 0.9% saline solution; 6 weeks after diabetes induction, nerve tissue samples harvested from the right sciatic nerve of rats in control (n = 3) and diabetic (n = 3) groups for transcription profiling.

GSE70852 using female black and tan, brachyuric background leptin deficient (BTBR ob/ob) mice display robust DPN, employed microarray technology to identify dysregulated genes and pathways in the SCN and DRG of female BTBR mice. Total RNA were extracted from DRG and SCN of 26 week old ob/+ and ob/ob mice (n = 5 in each group) and hybridized on Affymetrix microarray.

GSE155741 unveiled the effect of microcurrent electrical nerve stimulation (MENS) using omics data of mouse animal model. Mice were separated to 3 groups: control, STZ, STZ+MENS mice. STZ mice were treated with MENS for 6 consecutive weeks. RNA was prepared from liver and analyzed through microarray.

GSE156184 showed *in vitro* efficacy of alpha-lipoic acid (ALA) in the medication of symptomatic diabetic neuropathy, in the human prostate cell line of PSC27, as well as its efficacy in improvement of inflammatory diseases and diabetes induced by bleomycin.

Yu has generated protein profiling data of spinal dorsal horn using mass spectrometry (17), which was used to identify the differential expression of VGLUT2 in STZ-induced DPN rats (n = 12) and DPN rats with electro-acupuncture (EA) treatment (n = 12). The diabetes was induced by a single intraperitoneal injection of STZ (65 mg/kg, Sigma Chemicals, USA) dissolved in citrate buffer (10 mmol/L, Na citrate, pH = 4.3), and the acupoints “Zu sanli” and “Shen shu” were selected for stimulation. Needles were connected to a G6805-1A multifunctional EA apparatus (Shanghai Medical Electronic Apparatus Company, Shanghai, China), with a stimulation intensity of 1mA, frequency 10 HZ, 30 min/2 days, for four weeks.

### 2.9 Differential expression analysis

The R programming language was employed to extract expression values for the genes VGLUT2, APP, APLP1, and APLP2 through the online tool of GEO2R. GraphPad software 8 for Windows (GraphPad, La Jolla, CA, USA) was utilized for both statistical analysis and visualization of differential expression patterns. Statistical analysis was conducted following the procedures outlined in a previous study (15). All data are presented as means with the respective standard errors of the mean (SEMs) and were subjected to analysis using GraphPad Software, and Statistical Package for the Social Sciences (SPSS, version 22.0). Student’s t-test was employed to analyze and compare differences between the two groups of data, while one-way ANOVA was applied to analyze data with homogeneity of variance from multiple groups. A *p*-value < 0.05 was considered statistically significant. Each experiment was replicated at least three times.

### 2.10 Correlation analysis

Correlation analysis was conducted using data from the GEO database, where datasets from GSE were utilized for peripheral neural tissues associated with DPN and post-treatment DPN data from GSE. Expression values of key genes, including VGLUT2, APP, APLP1, and APLP2, were extracted and organized into a gene expression matrix. Coefficients for each pair of Y datasets were computed, resulting in a correlation matrix. A two-tailed analysis with a 95% confidence interval was performed, employing nonparametric Spearman correlation due to the assumption that the data were not sampled from a Gaussian distribution. Correlation significance was considered for values within the range -0.3 < *R* < 0.3 with a *p*-value < 0.05. GraphPad software was employed for the correlation analysis, generating a correlation matrix that was visualized as a heatmap.

### 2.11 Gene network construction

The Homo sapiens gene network was obtained from Gene MANIA. The input gene set comprised VGLUT2, APP family proteins, and NSC markers, resulting in the identification of 20 associated genes within the network. The connections in the network were characterized by shared protein domains, predicted interactions, physical interactions, pathways, co-localization, co-expression, and genetic interactions.

### 2.12 Functional enrichment analysis

The functional enrichment analysis of VGLUT2, APP, APLP1, and APLP2 was conducted using the STRING tool to discern Gene Ontology (GO) categories, specifically focusing on Biological Processes (BP). The inclusion of stemness markers such as NCAM1, NES, NGFR, and MSI1 augmented the input data for a comprehensive analysis. Visualization of the results was performed through Hiplot, accessible at https://hiplot.com.cn.

## 3 Results

### 3.1 The differential expression of VGLUT2 in DPN

**Figure 1:**
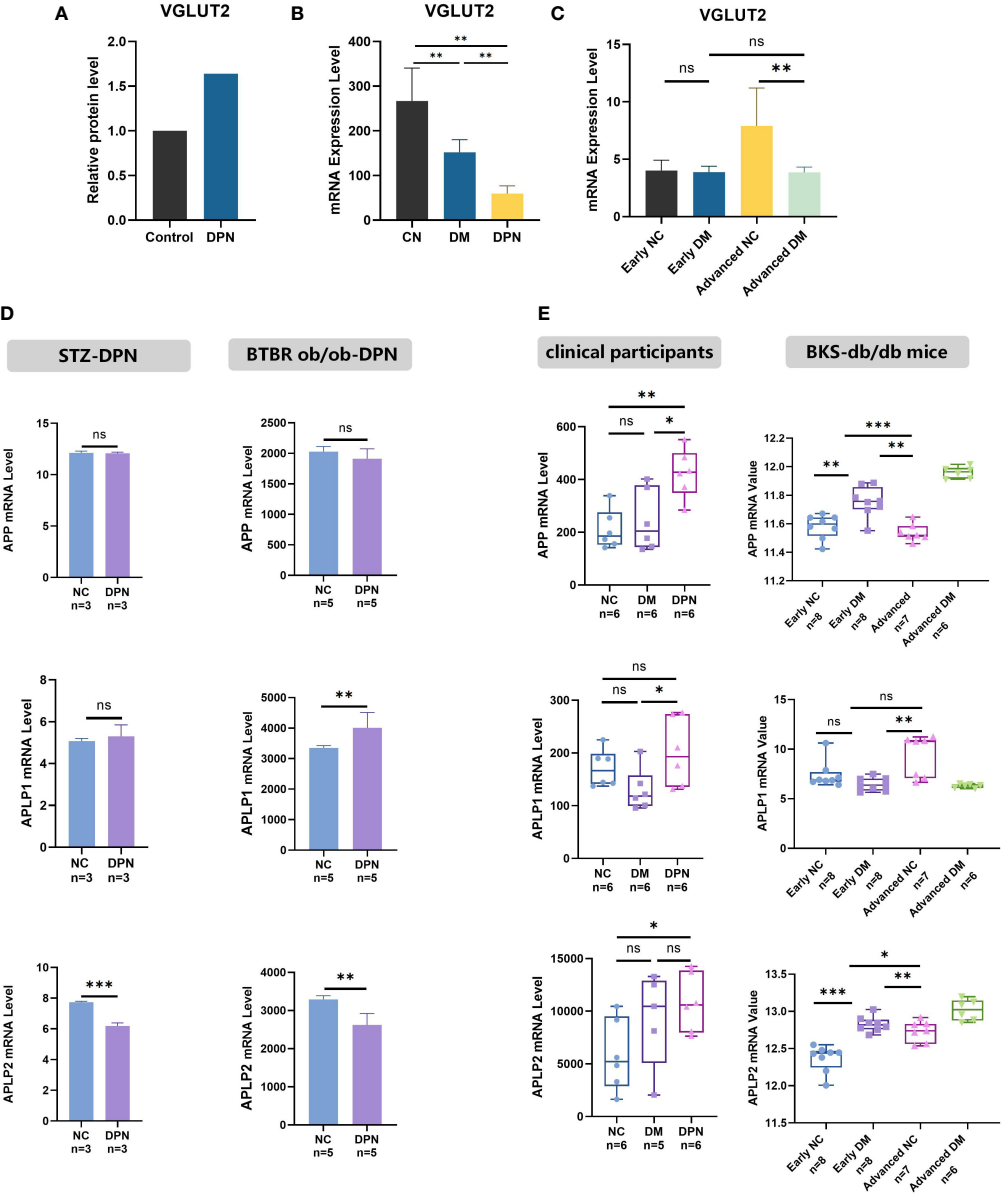
VGLUT2 and APP family gene differential expression in DPN. **(A)** The protein expression of DPN in the spinal dorsal horn of DPN rats, analyzed by protein mass spectrometry data. **(B, C)** The mRNA expression analysis for VGLUT2 in the blood sample of clinical participants (GSE95849) and in sciatic nerve of DPN mouse models constructed by BKS-db/db (GSE34889). **(D)** The mRNA expression analysis of APP, APLP1 and APLP2 in DPN was performed by microarray data from the sciatic nerve of STZ induced DPN rats (GSE147732) and the DRG of female BTBR ob/ob mice display robust DPN (GSE70852). **(E)** The expression of APP and related genes in stages of DPN was analyzed by microarray data from the blood sample of clinical participants (GSE95849) and the sciatic nerve of DPN mouse models constructed by BKS-db/db (GSE34889). NC, normal control; DPN, diabetic peripheral neuropathy; STZ, streptozotocin; BTBR-ob/ob, leptin deficient (black and tan, brachyuric background) mice; DM, diabetes mellitus; BKS-db/db, Cg-m+/+Lepr db/J (C57BLKS/J background) mice; MENS, microcurrent electrical nerve stimulation. *p-value < 0.05, **p-value <0.01, ***p-value < 0.001.

As shown in **Figure 1A**, VGLUT2 protein was upregulated with the fold change of 1.64 in DPN rats compared with normal ones (fold change = 1.64, *p-*value = 0.001939). In human blood, we found VGLUT2 significantly downregulated in DM patients, and even more decreased in DPN patients (**Figure 1B**). In the tissue of sciatic nerve, VGLUT2 expression declined with statistical significance in the advanced stage of DPN mouse models constructed by BKS-db/db, but there was no difference of gene expression in the early stage (**Figure 1C**). The differential expression in STZ-induced and BTBR ob/ob constructed DPN models was analyzed; the results indicated that APLP1 was elevated in dorsal root ganglia (DRG) only, whereas APLP2 was declined in both sciatic nerve and DRG, but there were no changes in APP expression (**Figure 1D**). Moreover, differences in APPs expression were observed according to histological disease progression and aging (**Figure 1E**). The above results suggest differential expression of APP and its related genes APLP1/2 in DPN, and their involvement in the disease progression.

### 3.2 The correlation between VGLUT2 and APPs varies in DPN

**Figure 2:**
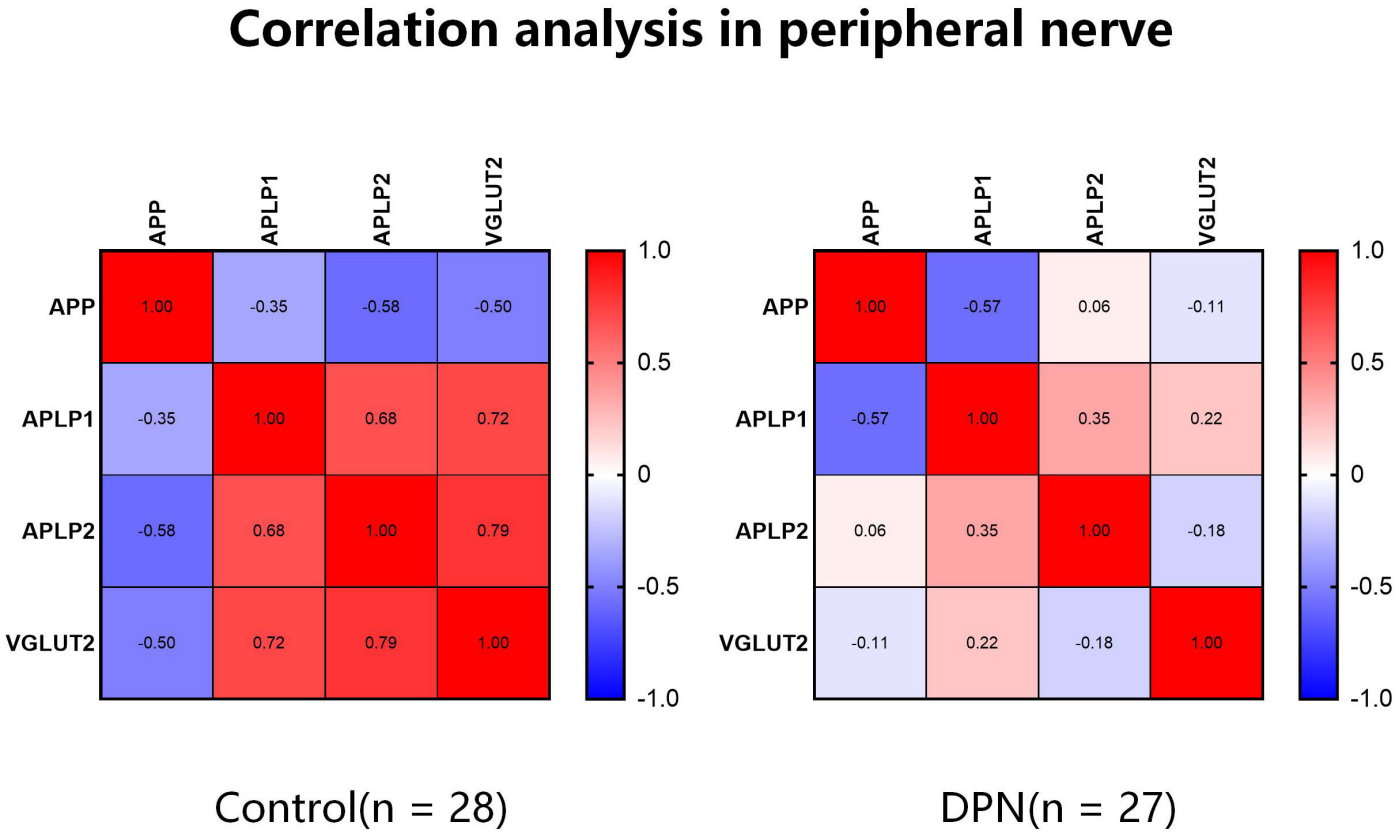
The correlation of VGLUT2 and APPs gene expression level varies between normal and diabetic peripheral nerve. Spearman correlation analysis was performed by mRNA expression data obtained from the GEO database, and the coefficients were visualized by heatmaps.

We further explored the role of APPs and VGLUT2 in DPN through Spearman correlation analysis. As shown in **Figure 2**, in normal peripheral nerve tissues (both sciatic nerve and DRG), VGLUT2 expression was negatively correlated with APP (*R* = -0.503, *p*-value = 0.034), while positively correlated with APLP1/2 (both *R* > 0.7, *p*-value < 0.001). Compared to the control group, the correlation between VGLUT2 and APPs in the DPN group was reduced, but the absolute values indicated no significant correlation between their gene expression levels (-0.3 < *R* < 0.3).

### 3.3 VGLUT2 and APPs up regulated in STZ-induced DPN mice

**Figure 3:**
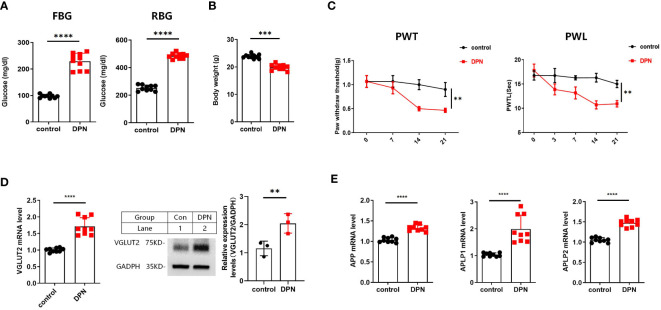
**(A)** FBG and RBG results from DPN mice induced by i.p. STZ 50mg/kg 4 times. **(B)** Body weight result from WT and DPN mice at 14 days after injection STZ. **(C)** STZ induces significant mechanical allodynia and thermal hyperalgesia in DPN mice hind paw. **(D)** The qPCR results for detecting VGLUT2 from spinal cord of WT and DPN mice at 14 days after STZ injection; and Western blot results from spinal cord of WT and DPN mice at 14 days after STZ injection for detecting VGLUT2 with the specific antibody. **(E)** The qPCR results for detecting APP, APLP1, and APLP2 from spinal cord of WT and DPN mice at 14 days after STZ injection. ** *p*-value < 0.01, *** *p*-value < 0.001, **** *p*-value < 0.0001, ANOVA. FBG, fasting blood glucose; RBG, random blood glucose; PWT, paw withdrawal threshold; PWL, paw withdrawal latency; WT, wild type.

The successful construction of the STZ-induced diabetes mellitus (DM) animal model was confirmed by a significant increase in FBG and RBG, as well as a significant decrease in body weight (**Figures 3A, B**). Subsequently, the successful establishment of the DPN mouse model was confirmed by PWT and PWL (**Figure 3C**). In the *in vivo* model of DPN, qPCR and western blot assays demonstrated a significant upregulation of VGLUT2 mRNA and protein expression in spinal dorsal horn (both *p*-value < 0.01) (**Figure 3D**). As shown in **Figure 3E**, the mRNA expression of APP, APLP1, and APLP2 significantly upregulated in DPN (*p*-value < 0.0001).

### 3.4 Electrical stimulation regulates VGLUT2 and APPs expression

**Figure 4:**
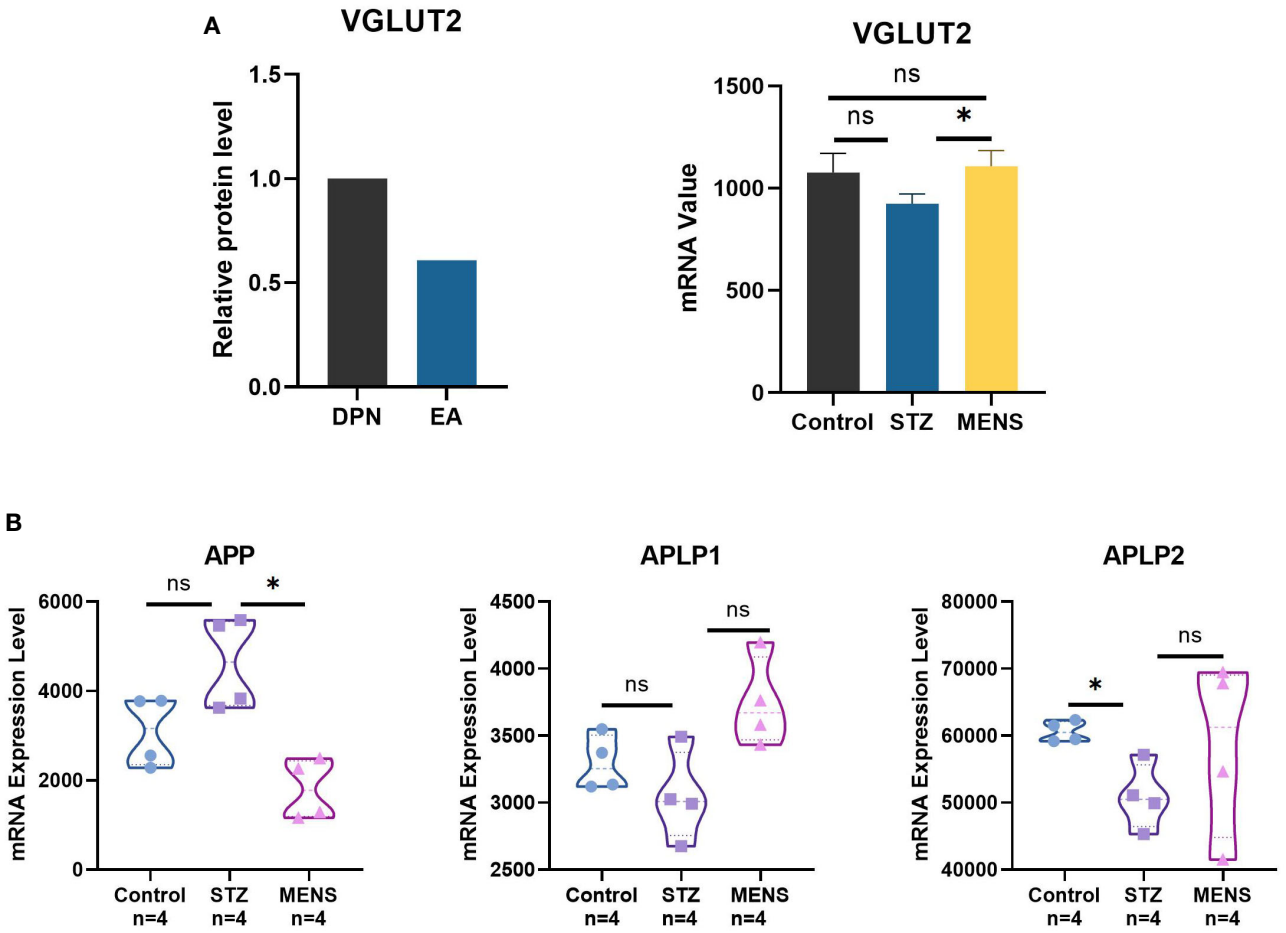
Neuroelectric stimulation therapy methods for DPN regulates the gene expression of VGLUT2 and APPs. **(A)** The protein level of VGLUT2 was qualified by protein profile for the spinal dorsal horn of DPN rat with EA treatment, and the mRNA level of VGLUT2 in livers of STZ-induced DPN mice and DPN mice with MENS treatment was qualified by GEO microarray (GSE155741). **(B)** The mRNA expression analysis of APP and related genes in DPN and MENS treatment was generated by GSE155741. * *p*-value <0.05, ns *p*-value > 0.05.

Compared with DPN rats, the protein level of VGLUT2 in DPN rats with EA treatment shows significant downregulation (**Figure 4A**) (fold change = 0.608, *p*-value = 9.99E-05). The mRNA expression of VGLUT2 significantly upregulated in liver of STZ-induced DPN mice with MENS treatment (*p* -value < 0.05). As shown in **Figure 4B**, the expression of APP significantly downregulated by MENS treatment (*p* -value < 0.05), but APLP1 and APLP2 in MENS rats did not show significant changes (*p* -value > 0.05). Compared with the control, APLP2 significantly downregulated in the DPN group, but no significant changes of APP and APLP1 were observed in STZ-induced DPN rats.

### 3.5 Prediction of DPN treatment mechanism based on VGLUT2 and APPs

**Figure 5:**
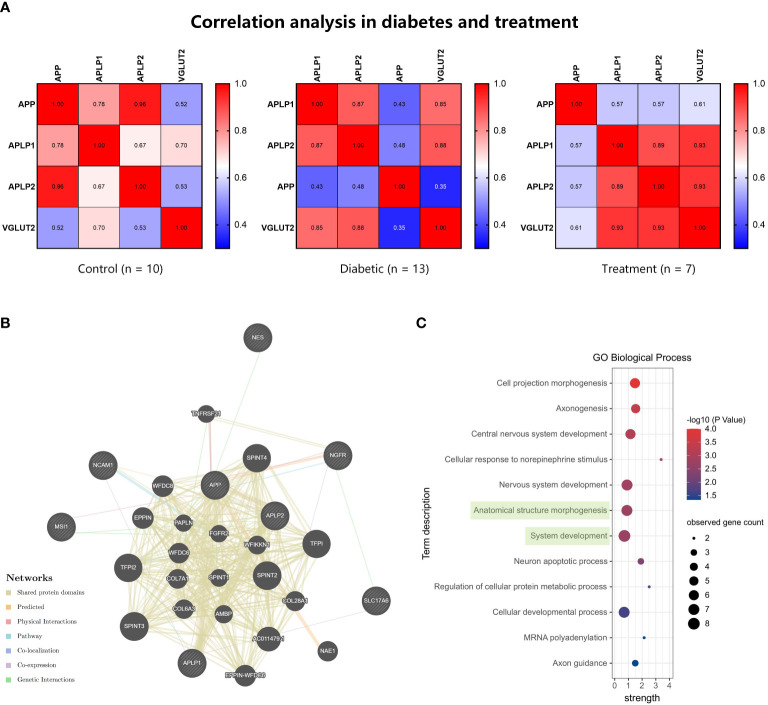
The gene function prediction analysis for VGLUT2 and APPs. **(A)** The Spearman correlation analysis was performed by mRNA expression data of VGLUT2, APP, APLP1, and APLP2 obtained from the GEO database. The heatmap showed the coefficients in normal, diabetic, and neuroelectric stimulation therapy tissues. **(B)** The construction of gene network based on Automatically selected weighting method in the GeneMANIA database. **(C)** The results of functional enrichment analysis in Biological Process, and terms including VGLUT2 were highlighted in green.

Spearman correlation analysis was performed for microarray data from GEO series (**Figure 5A**). In the DPN tissues treated with electrical stimulation, there was a positive correlation between the expression levels of VGLUT2 and APP and its associated protein genes. VGLUT2 exhibited a higher correlation with APLP1 and APLP2, but a relatively lower correlation with APP. Furthermore, considering the three conditions of control, DPN, and treatment, the correlation between VGLUT2 and APP showed an increasing trend in the disease state and a decreasing trend after treatment. The correlation between VGLUT2 and APLP1 showed a significant increase during DPN and a slight further increase after treatment. On the other hand, the correlation between VGLUT2 and APLP2 showed a decrease during the onset of DPN and a significant increase after disease treatment.

We constructed gene functional networks of these genes and neural stem cell factor (NGFR, NES, MSI1, NCAM1) using the GeneMANIA database. As shown in **Figure 5B**, these genes form a tightly interconnected network, with VGLUT2 exhibiting genetic interactions with the neurotrophic factor NGFR and co-expression with APLP1. Consistent with protein network analysis, functional enrichment analysis of VGLUT2, APPs, and stemness markers revealed significant associations in BP as well. The enrichment analysis results demonstrated that these genes were enriched in 12 BP terms, with VGLUT2 being associated with Anatomical structure morphogenesis (GO:0009653) along with NGFR, APLP1, APP, NES, NCAM1, and APLP2, and with System development (GO:0048731) along with NGFR, APLP1, MSI1, APP, NES, NCAM1, and APLP2 (**Figure 5C**).

## 4 Discussion

In previous studies, the transport of glutamate by VGLUT2 has been demonstrated as the rate-limiting step crucial for the precise regulation of insulin secretion, a process intricately linked to the onset and progression of diabetes. Additionally, in VGLUT2-cre mice generated through spared nerve injury, a notable reduction in the mechanical response threshold is observed (17). The downregulation of VGLUT2 expression in these mice is associated with an inhibition of the release of inflammatory factors, specifically TNF-α and IL-1β, leading to a relief in pain symptoms (18). Despite these findings, the expression and specific role of VGLUT2 in DPN remains poorly understood.

Through high-throughput data mining, we have discerned distinct expression patterns of VGLUT2 in various tissue types of DPN models. Notably, even within the realm of the nervous system, DPN exhibits varying regulatory effects on VGLUT2 in the central nervous system versus the peripheral nervous system. This expression result reflected that the differential expression of VGLUT2 in DPN was species and tissue-specific. Correlation analyses have unveiled a positive relationship between VGLUT2 and the amyloid protein family in gene expression. Given the pivotal role of APP and its associated genes in aging mechanisms, we postulate that the advanced stages of DPN predominantly modulate the gene expression of VGLUT2 in peripheral nerves at the transcriptional level. This mechanism offers a plausible explanation for the substantial upregulation of VGLUT2 in aging mice, as depicted in **Figure 1C**.

Recent investigations have illuminated the intricate relationship between AD and the onset and progression of diabetes. Shared risk factors for type 2 diabetes and AD encompass aging, obesity, and insulin resistance (19). Pharmacological studies suggest that certain traditional hypoglycemic drugs exert a discernible effect on AD treatment (20). While existing studies partially elucidate the potential link between Alzheimer’s disease and diabetes pathogenesis, the precise interaction mechanisms remain elusive. The role of APP gene mutations in AD is well-established, but this is not as well-explored in the context of Type 2 Diabetes Mellitus (T2DM). Intriguingly, investigations in middle-aged APdE9 mice have identified a diminished pre-synaptic glutamate response, potentially contributing to AD (12). Concurrently, VGLUT2 is closely associated with insulin transport and pain, offering a theoretical foundation for probing into the role of APP-VGLUT2 in DPN.

APLP1, APLP2, and APP are well-established substrates of β-site amyloid precursor protein cleaving enzyme 1 (BACE1) (21), playing crucial molecular roles in Alzheimer’s disease. The coexistence of Alzheimer’s disease and diabetes have garnered significant attention from both neuroscientists and researchers in metabolic disorders. Considering the regulatory roles of APP and APLP2 on VGLUT2 during neural differentiation, we conducted an investigation into APP and its associated genes. This work, we established a DPN animal model and confirmed a significant transcriptional upregulation of APP and its related genes in the spinal dorsal horn of STZ-induced mice through qPCR validation. Based on the results of our animal experiments, both VGLUT2 and APPs exhibited upregulated gene expression in the spinal dorsal horn tissue of DPN. To address this gap, we leveraged gene expression data obtained from GEO to scrutinize the correlation between VGLUT2 and the APP family gene expression within the peripheral neural tissues affected by DPN. In normative tissues, VGLUT2 manifested a negative correlation with APP and a positive correlation with APLP1/2. Although DPN samples exhibited no statistically significant correlation between VGLUT2 and the APP family, modifications in their correlation patterns in the context of DPN offer valuable insights for delving into the role of VGLUT2 in the mechanisms underpinning DPN development.

Data mining revealed differential expression of APP, APLP2, and APP in the DPN animal model, influenced by various factors, including DPN induction conditions, tissue types, disease progression, and aging. These findings suggest that the regulatory mechanisms of APPs in DPN may be intricate, influenced by multiple factors. However, this observed phenomenon lacked validation in peripheral nervous system (PNS) tissues, such as the sciatic nerve and DRG. Additionally, the gene expression regulation of these genes under different conditions awaits further confirmation, necessitating additional research into translational and post-translational levels.

Given the current ineffectiveness of clinical treatments for DPN, it is imperative to explore novel mechanisms and therapeutic approaches. In a meta-analysis of clinical studies investigating electroacupuncture treatment for DPN, four articles involving a total of 366 patients were included (22). The findings revealed that combining drug treatment with other acupuncture techniques or acupoint injections significantly enhanced the effectiveness rate compared to the use of western medicine alone. Consistent with these results, additional research has supported the notion that low-frequency electricity can inhibit the phosphorylation of Cav-1 in DRG, leading to a favorable analgesic effect in DPN. Moreover, electroacupuncture demonstrates the capacity to ameliorate Schwann cell apoptosis through the PI3K-AKT pathway (23), offering a potential therapeutic avenue for treating sciatic nerve injury in the context of DPN.

In our research, we analyzed the databases both EA and MENS as electrical stimulation therapies. Analysis of microarray data obtained from the liver tissues of mice with DPN undergoing microcurrent neurostimulation therapy indicates a selective modulation of specific genes within the APP family. Subsequent correlation analysis suggests that this therapeutic intervention can modify the relationship between VGLUT2 and the APP family, albeit with varying trends. Through protein-protein interaction analysis and functional enrichment analysis, we identify a noteworthy association between VGLUT2, APLP1, and NGFR. Furthermore, VGLUT2, in conjunction with the APP family and neural stem cell-related factors, assumes a significant role in the GO Biological Processes of anatomical structure morphogenesis and system development. We suggest that the differential expression of VGLUT2 after electrical stimulation were related to a variety of factors, among which omics differences, species and tissue specificity may be the main reasons.

Our investigation reveals that neurostimulation therapy, administered for the treatment of DPN, exerts regulatory effects on both the transcription and translation levels of the VGLUT2 gene. However, the trend of gene differential expression is subject to influences from variables such as the method of electrical stimulation, tissue type, and species. Drawing on a comprehensive examination of experimental results, we posit that both DPN and neurostimulation therapy can elicit differential expression in VGLUT2 and the APP family, indicating a certain level of correlation between these two phenomena. Future research will continue to focus on the molecular biological mechanism of APP family regulating VGLUT2, take the level of VGLUT2 gene expression as a biomarker of DPN, and then propose novel therapeutic methods based on VGLUT2 gene expression regulation.

## 5 Conclusion

This study, employing rigorous analyses in a STZ-induced DPN mouse model, unveiled distinctive expression patterns of VGLUT2 and the APP family, providing profound insights into the pathogenesis of DPN. Additionally, analysis of neurostimulation therapy revealed intricate differential expression patterns of VGLUT2 and APPs, predicting key biological processes involved in DPN development and therapy. This comprehensive study not only advances our understanding of DPN’s molecular intricacies but also lays a robust theoretical foundation for innovative and targeted neurostimulation therapy interventions.

## Data availability statement

Publicly available datasets were analyzed in this study. This data can be found here: http://www.ncbi.nlm.nih.gov/geo/.

## Ethics statement

The animal study was approved by Sun Yat-sen University Cancer Centre Animal Care and Use Committee (Sun Yat-sen University No.: L025501202205002). The study was conducted in accordance with the local legislation and institutional requirements.

## Author contributions

YZ: Writing – original draft, Data curation, Funding acquisition, Visualization, Writing – review & editing. CW: Formal Analysis, Investigation, Software, Writing – original draft. WJ: Methodology, Writing – original draft. YC: Investigation, Methodology, Project administration, Writing – original draft, Writing – review & editing. DC: Project administration, Supervision, Writing – original draft, Writing – review & editing.
